# Knowledge, attitude, and practices toward infection prevention and control among undergraduate pharmacy students in Zambia: findings and implications

**DOI:** 10.1017/ash.2023.428

**Published:** 2023-09-11

**Authors:** Steward Mudenda, Joseph Chizimu, Billy Chabalenge, Maisa Kasanga, Scott Kaba Matafwali, Victor Daka, Kaunda Yamba, Margret Mulomba, Webrod Mufwambi, Patricia Katowa-Mukwato, Brian Godman

**Affiliations:** 1 Department of Pharmacy, School of Health Sciences, University of Zambia, Lusaka, Zambia; 2 Antimicrobial Resistance Coordinating Committee (AMRCC), Zambia National Public Health Institute, Lusaka, Zambia; 3 Department of Medicines Control, Zambia Medicines Regulatory Authority, Lusaka, Zambia; 4 Zhengzhou University, College of Public Health, Zhengzhou, Henan, China; 5 Clinical Research Department, Faculty of Infectious and Tropical Diseases, London School of Hygiene & Tropical Medicine, London, UK; 6 Department of Public Health, Michael Chilufya Sata School of Medicine, Copperbelt University, Ndola, Zambia; 7 Department of Basic and Clinical Nursing, School of Nursing Sciences, University of Zambia, Lusaka, Zambia; 8 Department of Public Health Pharmacy and Management, School of Pharmacy, Sefako Makgatho Health Sciences University, Pretoria, South Africa; 9 Centre of Medical and Bio-allied Health Sciences Research, Ajman University, Ajman, United Arab Emirates; 10 Department of Pharmacoepidemiology, Strathclyde Institute of Pharmacy and Biomedical Science (SIPBS), University of Strathclyde, Glasgow, UK

**Keywords:** Infection prevention practices, nosocomial infections, antimicrobial resistance, pharmacy students, Zambia

## Abstract

**Background:**

Infection prevention and control (IPC) measures are critical in preventing the risk of acquiring and transmitting nosocomial infections. In Zambia, there is little information concerning IPC practices among pharmacy students who are exposed to potentially infectious areas both in public and private healthcare settings. Therefore, this study assessed the knowledge, attitude, and practices of undergraduate pharmacy students toward infection prevention and control at the University of Zambia.

**Materials and methods:**

This cross-sectional study was conducted among 290 undergraduate pharmacy students at the University of Zambia using a structured questionnaire from August 2022 to October 2022. Data analysis was performed using SPSS version 25.0, and statistical significance was set at a 95% confidence level.

**Results:**

Of the 290 participants, 166(57.2%) were female and the majority were aged between 18 and 23 years. Overall, 252(86.9%) had good knowledge, 167(57.6%) had positive attitudes, and 248(85.5%) had good practices toward IPC measures. These results indicate lower attitude scores compared to knowledge and practices. Having good knowledge of IPC was associated with being a Christian by religion compared to other religions (OR = 5.314, 95% CI: 1.141–24.745). There was no association between sociodemographics and attitude and practice concerning IPC.

**Conclusion:**

This study found that pharmacy students had good knowledge, positive attitudes, and good practices toward IPC measures. Consequently, more emphasis is needed to improve the student’s knowledge, attitudes, and practices toward IPC, especially in areas where gaps were identified. Additionally, there is a need to improve curricula on IPC measures in the training of pharmacy programs.

## Background

Healthcare-associated infections (HAIs), also known as nosocomial infections, are infections that individuals acquire during their stay in or visit to healthcare facilities.^1^ HAIs also include infections that appear after hospital discharge and occupational infections among healthcare workers (HCWs) and healthcare students.^2^ Infection prevention and control (IPC) groups provide strategies that should be used across sectors to minimize the risk of infection transmission in healthcare settings.^3^ IPC has been defined as procedures, activities, and policies aimed at preventing or minimizing the risks of infection transmission in healthcare facilities.^4^ Due to high rates of HAIs, particularly across Africa and Asia,^5–8^ and as a result of them being among the ten threats to global health,^9^ there is a need to instigate effective IPC practices across sectors.

The lack of IPC measures in hospitals is a contributing factor to increased HAIs and antimicrobial resistance (AMR).^10^ Infections cause increased morbidity and mortality globally, especially drug-resistant infections.^11^ Other consequences of drug-resistant infections include increased medical costs and a negative impact on the country’s economy.^11^ As a result, the prevention of HAIs is critical and must be supported by practical and evidence-based methods, thereby decreasing their adverse socioeconomic and psychological impact.^4^ Comprehensive programs and policies are the cornerstones of resilient healthcare systems’ effectiveness in the prevention, detection, and response to public health emergencies including disease outbreaks and HAIs.^1,12^

IPC measures focus on how infections are transmitted and include standard contact, droplet, and airborne precautions.^13^ Standard precautions include the use of appropriate personal protective equipment (PPE) and hand hygiene, as well as employing aseptic procedures that prevent contact with microorganisms.^1,14^ Alongside this, the appropriate management of used needles, blood spills, linen, and waste is necessary to ensure a safe environment.^15^ Given this, compliance with agreed safety protocols by healthcare students as part of their training is an effective strategy to prevent and control HAIs.^13^ Consequently, there should be stipulated guidelines, teams, training, monitoring, and surveillance of IPC within healthcare facilities, alongside appropriate input in academic curricula, to enhance adherence to agreed practices.^1^

To date, studies undertaken in Africa have demonstrated inconsistencies in the knowledge, attitudes, and practices of HCWs toward IPC.^2^ Alongside this, few studies in Africa have reported on the knowledge, attitude, and practices of health sciences students concerning IPC. This is important for students who are the next generation of HCWs. A study in Namibia among health science students reported that the students were required to be taught IPC measures before being introduced to clinical practice,^16^ which improves knowledge in practice.^16–18^ This is because training on IPC equips students with skills and knowledge on how to prevent HAIs, translating into reduced risk and frequency of infections in practice.^19,20^

IPC practices are crucial in preventing further transmission and spread of the coronavirus disease 2019 (COVID-19).^3,21^ Studies have shown that IPC measures were among the recommended prevention measures to contain the pandemic by the World Health Organization (WHO).^21–23^ Some of the IPC measures that were recommended during the pandemic include wearing facemasks, hand hygiene, and wearing PPE.^21,22^ Evidence has also shown that COVID-19 led to an improvement in adherence to prevention measures no doubt assisted by fears of catching COVID-19 without such measures.^24^ However, other authors have found inconsistencies in the adherence and compliance to IPC measures during the pandemic.^25^ Alongside this, there are concerns about gaps in knowledge, attitudes, and practices toward IPC measures during the COVID-19 pandemic.^26^ These inconsistencies could have been caused by a lack of IPC resources, inadequate hospital infrastructure, lack of training on IPC, increased workload, shortage of HCWs, increased number of visitors, and increased disease burden alongside HCW burnout.^10^

Zambia is a country in sub-Saharan Africa that is affected by a high burden of infectious diseases, including HIV, TB, malaria, and respiratory infections incorporating the current COVID-19.^22,27–29^ Consequently, this calls for strengthening IPC measures in healthcare facilities.^30^

Health sciences students, including pharmacy students, are at increased risk of contracting HAIs because they are introduced to hospital practice during their training.^16^ However, to the best of our knowledge, there are currently no published studies on IPC practices among pharmacy students in Zambia. This study assessed the knowledge, attitudes, and practices of undergraduate pharmacy students toward IPC at the University of Zambia.

## Materials and methods

### Study design, site, and population

We conducted a cross-sectional study at the University of Zambia among undergraduate pharmacy students from August 2022 to September 2022. The Bachelor of Pharmacy degree program is offered under the School of Health Sciences at Ridgeway campus in Lusaka, Zambia. To be eligible, a student had to be enrolled in the Bachelor of Pharmacy degree program and should have provided consent to be a participant.

### Sample size determination and sampling criteria

The target population included all undergraduate pharmacy students at the Ridgeway campus. The enrolled students in the Bachelor of Pharmacy program were 593 in total that included 195 second years, 170 third years, 103 fourth years, and 125 fifth years. Employing a margin of error of 5%, we used Tora Yamane’s formula to estimate the required sample size, resulting in a sample size of 239. We took into consideration a non-response rate of 10%, and this translated into a minimum sample size of 263. Consequently, factoring in proportions according to population size, we required a minimum sample size of 86 second-year, 75 third-year, 46 fourth-year, and 56 fifth-year students. We subsequently distributed 300 questionnaires to the potential participants who were selected using a simple random sampling method.

### Data collection

We collected the data using a structured questionnaire which was adapted from a previous study.^31^ The data collection tool had four sections. These included Section A, which had questions on the sociodemographic characteristics of the participants; Section B, which had questions on the knowledge of participants on IPC; Section C, which had questions on the attitudes of participants toward IPC; and Section D, which had questions on the practices of participants toward IPC. We subsequently conducted a pilot study among 30 students drawn from the Biomedical Sciences department to add robustness to the questionnaire. The results from the pilot study were used to optimize the data collection tool for logic and consistency and were excluded from the final analysis for the study. Data collection was performed by two data collectors and took approximately 20–30 minutes per participant to fill in the questionnaire. A five-point Likert scale was used to assess the student’s knowledge, attitudes, and practices regarding IPC.

### Data analysis

The data that were collected were entered into Microsoft Excel (Microsoft Corp., Redmond, WA) for cleaning. The data were then coded and entered into Statistical Package for the Social Sciences (SPSS) version 25.0 for analysis. In the analysis, strongly agree was assigned a score of 5, agree a score of 4, neutral a score of 3, disagree a score of 2, and strongly disagree a score of 1. Knowledge questions were four, translating into a total score of 20, while attitude questions were five, resulting in a total score of 25. There were four practice questions, which meant a total score of 20. Good KAP concerning IPC was considered to be scores of 70% and above (scores of 14 and above for knowledge and practices while scores of 17.5 and above for attitudes). Descriptive statistics were performed on the sociodemographic characteristics, and the results were presented in the form of frequencies and percentages in tables. Univariate analysis was used to determine the relationships between KAP scores and sociodemographic characteristics. All the characteristics that had *p* < 0.25 were taken to build the model in binary logistic regression. The goodness of fit was determined using the Hosmer and Lemeshow test. In the final model, all factors that had a *p* < 0.05 were considered statistically significant at a 95% confidence level and were associated with the students’ KAP on IPC. The odds ratios (OR) and 95% confidence intervals (95% CI) were reported.

### Ethical approval

Ethical approval was granted by the University of Zambia Health Sciences Research Ethics Committee (UNZAHSREC) with protocol ID #: 2022112301179. All participants were informed about the purpose of the study, and they all provided informed consent before responding to the questionnaire.

## Results

### Sociodemographics of study participants

This study enrolled 290 pharmacy students giving a response rate of 97%, with 57.2% being female and the majority aged between 18 and 23 years (Table 1).

**Table 1.** Sociodemographic characteristics of participants

Variable	Attribute	Frequency	Percent	*p*-value
Gender	Female	166	57.2	0.016
	Male	124	42.8	
Age (years)	18–23	146	50.3	0.001
	24–29	117	40.3	
	30–35	21	7.2	
	Above 35	6	2.1	
Religion	Christianity	283	97.6	0.001
	Islam	4	1.4	
	Others	3	1.0	
Year of Study	Second	86	29.7	0.119
	Third	79	27.4	
	Fourth	62	21.4	
	Fifth	63	21.7	

Most students (31.7%) thought that practicing hand hygiene using alcohol-based rubs was preferable to handwashing with soap; however, almost the same percentage (29.7%) disagreed (Table 2).

**Table 2.** Participants’ knowledge of infection prevention and control

Knowledge questions	Attribute	Frequency	Percent	*p*-value
Hand hygiene with alcohol-based rubs is always preferred over soap and water	Strongly disagree	29	10.0	0.001
	Disagree	86	29.7	
	Agree	92	31.7	
	Strongly agree	55	19.0	
	Neutral	28	9.7	
Hospital-acquired infections are transmitted during close contact and through droplets	Strongly disagree	5	1.7	0.001
	Disagree	8	2.8	
	Agree	144	49.7	
	Strongly agree	108	37.2	
	Neutral	25	8.6	
A person can be infected with bacteria or viruses by touching surfaces where droplets fall	Strongly disagree	6	2.1	0.001
	Disagree	6	2.1	
	Agree	148	51.0	
	Strongly agree	119	41.0	
	Neutral	11	3.8	
Medical masks should be used when entering the hospital premises	Strongly disagree	9	3.1	0.001
	Disagree	10	3.4	
	Agree	105	36.2	
	Strongly agree	161	55.5	
	Neutral	5	1.7	

Some students (45.5%) felt that were adequately prepared to attend to patients suffering from infectious diseases; however, 41% did not feel safe interacting with patients (Table 3).

**Table 3.** Participants’ attitudes toward infection prevention and control

Attitude questions	Attribute	Frequency	Percent	*p*-value
I am adequately prepared to attend to patients suffering from infectious diseases	Strongly disagree	12	4.1	0.001
	Disagree	37	12.8	
	Agree	132	45.5	
	Strongly agree	41	14.1	
	Unsure	68	23.4	
I would wear the required personal protective equipment even if it is uncomfortable	Strongly disagree	16	5.5	0.001
	Disagree	21	7.2	
	Agree	144	49.7	
	Strongly agree	99	34.1	
	Unsure	10	3.4	
I feel safer using an alcohol-based hand rub than washing my hands with soap and water	Strongly disagree	37	12.8	0.001
	Disagree	101	34.8	
	Agree	81	27.9	
	Strongly agree	55	19.0	
	Unsure	16	5.5	
I feel safer interacting with patients with infectious diseases even if the required personal protective equipment is not available	Strongly disagree	118	40.7	0.001
	Disagree	119	41.0	
	Agree	16	5.5	
	Strongly agree	10	3.4	
	Unsure	27	9.3	
I feel that I could be infected with a bacterial or viral disease in the hospital regardless of the precautions I take	Strongly disagree	24	8.3	0.001
	Disagree	79	27.2	
	Agree	125	43.1	
	Strongly agree	21	7.2	
	Unsure	41	14.1	

Encouragingly, most students (53.4%) practiced handwashing regularly to prevent acquiring infections. Additionally, 43.1% wore facemasks and 45.9% wore closed shoes when in the hospital environment (Table 4).

**Table 4.** Participants practice questions regarding infection prevention and control

Practice questions	Attribute	Frequency	Percent	*p*-value
I wash my hands regularly to minimize the chances of acquiring infections	Strongly disagree	6	2.1	0.001
	Disagree	17	5.9	
	Agree	155	53.4	
	Strongly agree	103	35.5	
	Unsure	9	3.1	
I wash my hands for about 30 seconds and longer	Strongly disagree	17	5.9	0.001
	Disagree	36	12.4	
	Agree	132	45.5	
	Strongly agree	51	17.6	
	Unsure	54	18.6	
I wear a facemask when entering the hospital premises	Strongly disagree	6	2.1	0.001
	Disagree	27	9.3	
	Agree	125	43.1	
	Strongly agree	125	43.1	
	Unsure	7	2.4	
I wear closed shoes when I am on the hospital premise	Strongly disagree	4	1.4	0.001
	Disagree	23	7.9	
	Agree	133	45.9	
	Strongly agree	122	42.1	
	Unsure	8	2.8	

Overall, pharmacy students had good KAP concerning IPC practices with females recording better scores than their male counterparts (Table 5).

**Table 5.** Overall KAP of students on IPC

Variable	Frequency (%)	Females	Males
Knowledge	252(86.9)	146	106
Attitudes	167(57.6)	102	65
Practices	248(85.5)	142	106

Christians were also more likely to have good knowledge of IPC than other religious groups (OR = 5.314, 95% CI: 1.141–24.745) (Table 6).

**Table 6.** Factors affecting KAP toward IPC among pharmacy students

Variable	Characteristics	Univariate analysis (*p*-value)	Attributes	OR (95% CI)	*p*-value
Knowledge	Gender	0.599	Male	Ref	0.534
			Female	–	
	Age	0.064	30 years and above	Ref	0.069
			18–29 years	–	
	Religion	0.040	Other regions	Ref	0.003
			Christianity	5.314(1.141–24.745)	
	Year of Study	0.483	Clinical year students	Ref	0.461
			Preclinical students	–	
Attitude	Gender	0.150	Male	Ref	0.124
			Female	–	
	Age	0.840	Ref 30 years and above	Ref	0.823
			18–29 years	–	
	Religion	0.139	Other regions	Ref	0.116
			Christianity	–	
	Year of Study	1.000	Clinical year students	Ref	0.997
			Preclinical students	–	
Practice	Gender	1.000	Male	Ref	0.989
			Female	–	
	Age	1.000	30 years and above	Ref	0.959
			18–29 years	–	
	Religion	1.000	Other regions	Ref	0.988
			Christianity	–	
	Year of Study	0.739	Clinical year students	Ref	0.710
			Preclinical students	–	

## Discussion

To the best of our knowledge, this was the first study to assess the pharmacy students’ KAP concerning IPC in Zambia. We found that most students had good knowledge (86.9%), positive attitudes (57.6%), and good practices (85.5%) toward the IPC measures. Having good knowledge of IPC was also associated with being a Christian by religion.

Good knowledge of IPC measures among pharmacy students in Zambia mirrors findings from India,^32^ Saudi Arabia,^17^ Malaysia,^33^ South Africa,^18^ and Uganda,^31^ where most students had good knowledge of IPC practices. Good knowledge could be due to the knowledge students acquire during their training, potentially enhanced by the recommendations regarding IPC measures for all populations during the COVID-19 pandemic by the WHO.^23^ Additionally, a study in Switzerland found that increased knowledge and adherence to IPC measures were observed during the COVID-19 pandemic.^24^ Overall, building on the lessons learnt from the COVID-19 pandemic, there is typically a need to improve students’ knowledge regarding IPC through educational training and workshops.^34^ There is also a need to promote behavioral change toward IPC among students, given its importance.^35^

Most of the students in our study thought that alcohol-based hand rubs were better than handwashing with soap to prevent infections. Conversely, this was followed by a group that felt that handwashing with water and soap was preferable to hand rubs. Overall, handwashing has been highly practiced as a disease-preventive measure by students, as reported by other studies.^36,37^ Hand hygiene remains a critical component of IPC measures in healthcare facilities across the globe.^1,3^

Encouragingly, the majority of pharmacy students in our study had positive attitudes toward IPC. Having said this, compared to the knowledge scores, the attitude scores of the students on IPC were lower. This, though, is similar to a study that was conducted among medical students in Sri Lanka, where most had positive attitudes toward IPC measures.^38^ However, a study in South Africa found contrasting results in which most nursing students had negative attitudes toward IPC.^18^ This is a concern as negative attitudes toward IPC may predispose individuals to infections, especially HAIs. Consequently, where concerns exist, there is a need to improve the college and university curriculum concerning IPC measures^16^ and the students’ attitudes.^39^

Encouragingly as well, most pharmacy students in our study had good self-reported practices toward IPC. This is in line with a study that was conducted among nursing students which found good self-reported practices toward IPC measures.^40^ The good practices concerning IPC among students could be due to their training and experiences to adhere to the COVID-19 prevention measures during the pandemic. Conversely, a study in India reported sub-optimal practices toward IPC among medical students.^32^ Subsequently, a study in Ghana found that the majority of medical students had poor practices toward handwashing despite having received training and being knowledgeable about it.^37^ The poor practices regarding IPC among students are a public health concern that requires urgent educational interventions and behavioral change to increase the uptake of IPC measures going forward, with subsequent monitoring of future activities.^35^ This is particularly important during pandemics, especially among African countries, where there are real concerns about AMR. It is crucial to reduce HAIs in these countries due to the significant implications AMR can have when managing such infections.

We are aware of some limitations of this study. Firstly, it was conducted at only one institution of higher learning in Zambia. In addition, the questionnaire was adapted from a previous study. However, we undertook a pilot study to help address this limitation. Overall, despite these limitations, we believe our findings are robust enough to be a foundation for future research and provide educational policy direction for future designs.

## Conclusion

This study demonstrated that undergraduate pharmacy students in Zambia had good knowledge, positive attitudes, and good practices toward IPC during the COVID-19 pandemic. However, there is a need to provide IPC awareness programs to students and graduates with an emphasis on areas where gaps were found. Finally, the curriculum for pharmacy training must be improved in the areas of IPC. This is critical in reducing the burden of infectious diseases in Zambia and improving the use of antimicrobials in the future. As a result, this may reduce the current burden of AMR in Zambia and its associated impact on morbidity and mortality.

## Data Availability

Data can be made available on request from the corresponding author.
